# Case Report: Development of severe inflammatory orbitopathy after immune checkpoint inhibitor initiation

**DOI:** 10.3389/fopht.2025.1574643

**Published:** 2025-06-03

**Authors:** Samantha Madala, Alomi Parikh, Kendra Hong, Michael Burnstine

**Affiliations:** ^1^ University of Southern California (USC) Roski Eye Institute, Keck School of Medicine of the University of Southern California, Los Angeles, CA, United States; ^2^ Eyesthetica, Oculofacial and Cosmetic Surgery Associates, Los Angeles, CA, United States

**Keywords:** immune checkpoint inhibitor, inflammatory orbital disease, thyroid eye disease (TED), thyroid eye disease treatment, PD-1 inhibitor, CTLA-4 inhibitor

## Abstract

We report a case of thyroid eye disease (TED) reactivation, review the literature, and raise awareness of severe ophthalmopathy and myositis following the initiation of cancer treatment with an immune checkpoint inhibitor (ICI). In this case, after starting nivolumab, a 68-year-old woman with a past medical history of stage 2C uterine carcinoma status post-hysterectomy and past ocular history of thyroid eye disease developed ophthalmoplegia, proptosis, decreased color vision, optic disc hemorrhage, and ocular inflammation. Halting nivolumab and starting 2 weeks of intravenous steroids, one dose of teprotumumab, and low-dose orbital radiation resulted in an improvement in her orbitopathy. ICIs have become popular in oncologic treatment regimens, but they can have serious adverse effects including thyroiditis, Hashimoto’s hypothyroidism, or Graves’ disease complicated by TED. Multiple cases have been reported in the literature of both the reactivation of TED and the presentation of a TED-like orbital inflammation, which we summarize here. Awareness of orbital inflammation due to ICI use is critical, and ICIs should be used with caution in patients with a history of thyroid disease due to the risk of TED reactivation.

## Introduction

In recent years, immune checkpoint inhibitors (ICIs) have become a mainstay of cancer management. ICIs are currently used as adjuvant and neo-adjuvant therapies as well as primary chemotherapy for metastatic cancers and have been approved to treat over 20 types of tumors thus far (1). Like all immunotherapies, ICIs like ipilimumab and tremelimumab, which target cytotoxic T-lymphocyte-associated protein 4 (CTLA-4), and nivolumab and pembrolizumab, which target programmed cell death protein 1 (PD-1), are associated with adverse effects, including hypothyroidism, Graves’ disease, and painless thyroiditis (2–4). Herein, we describe a case of severe ophthalmopathy following initiation of cancer treatment with nivolumab and outline previous cases of similar orbital inflammation that coincided with ICI use. Written informed consent was obtained from the patient for the publication of this information in accordance with the Declaration of Helsinki.

## Case report

**Figure 1:**
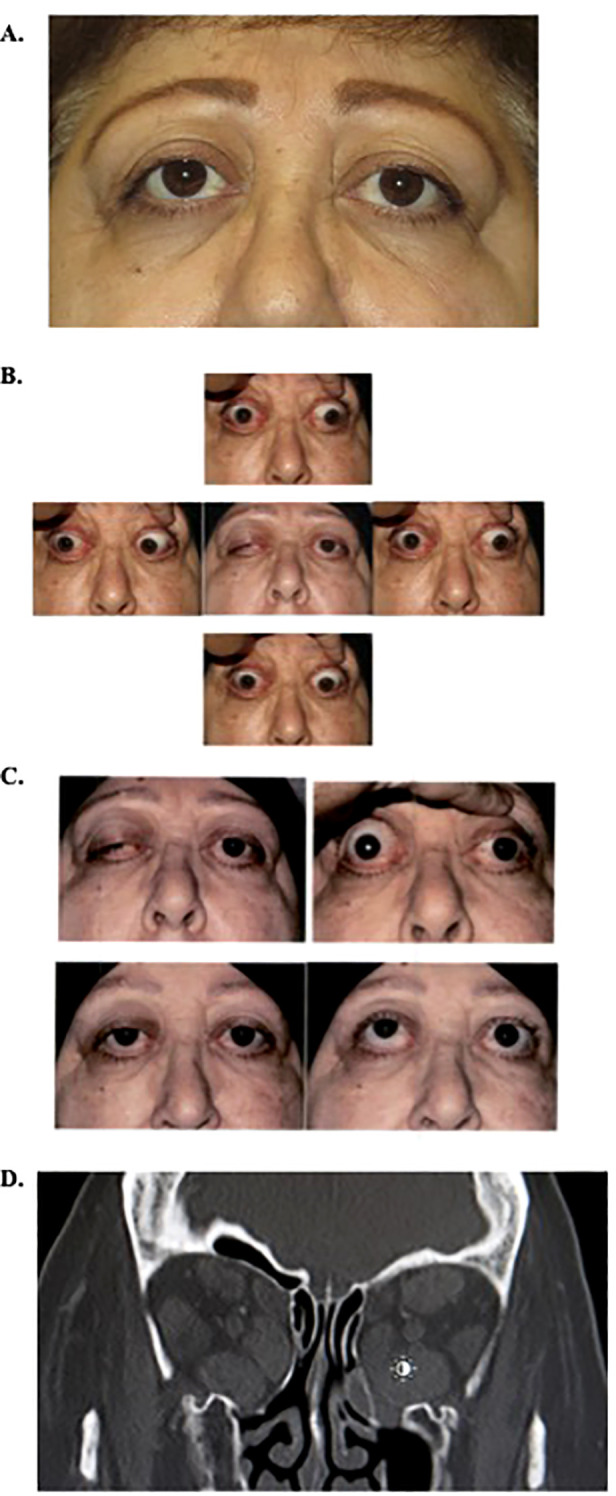
**(A)** External photo prior to the current episode of TED reactivation, after bilateral graded transantral decompression in 2004, Clinical Activity Score of 0. **(B)** External photos on initial presentation showing reactivation of TED demonstrating ptosis, proptosis, and decreased extraocular motility in all fields of gaze. **(C)** External photos before and 4 weeks after initial presentation following IV methylprednisolone, one dose of teprotumumab, and two doses of low-dose orbital radiation. Photos in primary and upgaze. **(D)**. Coronal image, computed tomography of orbits on current presentation, demonstrating enlargement of extra-ocular muscles. Note previous graded transantral orbital decompression with infracture of medial wall and orbital floor removal. TED, thyroid eye disease.

The patient is a 68-year-old woman with a past medical history of stage 2C uterine carcinoma status post-hysterectomy and lymph node dissection 3 months prior and past ocular history of thyroid eye disease (TED) s/p bilateral transantral orbital decompression (2004) (**Figure 1A**) and teprotumumab infusion (complete cycle of eight infusions in 2023). She presented with 1 week of double vision, proptosis, and increasingly severe bilateral orbital pain after two cycles of chemotherapy with nivolumab for endometrial cancer management. On exam, her visual acuity was 20/25 on the right and 20/20 on the left, intraocular pressure was normal, and pupils were round and reactive bilaterally with no afferent pupillary defect. She had right-sided color desaturation, measured using Ishihara color plates. Her extraocular movements were −4 in all directions, and she exhibited proptosis (Hertel exophthalmometry base 100; eye protrusion from orbital rim 30 mm OU) and restrictive right upper eyelid ptosis (**Figure 1B**). The external exam was remarkable for brow fullness and edema, eyelid swelling, lid lag, conjunctival injection, caruncle inflammation, and erythema [Clinical Activity Score (CAS) 5]. A dilated funduscopic exam revealed a right-sided inferior splinter disc hemorrhage. Computed tomography of the head revealed enlargement of extraocular muscle bellies sparing the muscle tendons bilaterally, as well as taut optic nerves without globe tenting bilaterally, consistent with thyroid eye disease rather than infection or metastasis (**Figure 1D**). Her chemotherapy was stopped, and she received multiple doses of intravenous methylprednisolone of 1.25 g and was referred to radiation oncology for low-dose orbital radiation. On a follow-up visit, she presented with dysphagia of unclear etiology and was admitted to the hospital for a higher level of care where she received one dose of teprotumumab for new-onset left optic neuropathy. At 1 month after presentation, the combination of 2 weeks of IV steroids, one dose of teprotumumab, and two doses of 2-Gy photonic orbital radiation led to improvement in her eyelid swelling and erythema, decreased lid lagophthalmos, reduced conjunctival injection, disappearance of the disc hemorrhage, resolution of her orbital pain, and improvement in extraocular movements. Her proptosis decreased (Hertel exophthalmometry base 100; eye protrusion from orbital rim 25 mm OD and 26 mm OS; Clinical Activity Score 1; **Figure 1C**). She opted to forgo any additional chemotherapy for her uterine cancer.

## Previous reports of orbital inflammation

Along with this case, there are rare case reports of a direct link between ICI use and the reactivation of TED, including those resulting in vision loss (**Table 1**). Similar to our reported case, multiple patients experienced severe reactivation of thyroid eye disease with nivolumab, a PD-1 inhibitor. While one patient developed eyelid retraction and ophthalmoplegia with minimal inflammation amounting to a CAS of 1 and thyroid function testing consistent with a hyperthyroid state, other patients developed more active inflammatory orbitopathies without any abnormalities in thyroid function testing. None of these patients had a prior history of thyroid eye disease, but the majority demonstrated diffuse enlargement of all extraocular muscles on imaging (5–7).

**Table 1:** Cases of thyroid eye disease after immune checkpoint inhibitors.

Publication	Checkpoint inhibitor	Key exam findings	Intervention	Outcome
“Thyroid-like ophthalmopathy in a euthyroid patient receiving Ipilimumab” (2014) (9)	Ipilimumab	Proptosis and ophthalmoplegia bilaterally	IV methylprednisolone followed by oral prednisolone	Improvement in extraocular movements
“Drug-induced Graves Disease from CTLA-4 receptor suppression” (2011) (12)	Ipilimumab	Severe proptosis, ophthalmoplegia, and exposure keratitis	Cantholysis and corticosteroids	Resolution of orbital inflammation
“Severe Inflammatory Ophthalmopathy in a Euthyroid Patient during Nivolumab Treatment” (2018) (5)	Nivolumab	Bilateral ptosis, conjunctival injection and chemosis, proptosis, and ophthalmoplegia	IV methylprednisolone	Improvement in extraocular movements and proptosis
“Extraocular Muscle Enlargement and Thyroid Eye Disease-like Orbital Inflammation Associated with Immune Checkpoint Inhibitor Therapy in Cancer Patients” (2019) (6)	Nivolumab	Periocular pain, pain with eye movements, ocular irritation, eyelid swelling and erythema, and ophthalmoplegia	IV methylprednisolone followed by oral prednisolone	Improvement in inflammation
	Nivolumab	Lid retraction and ophthalmoplegia without inflammatory signs	Observation	Gradual improvement in ophthalmoplegia
	Tremelimumab	Periocular swelling and erythema with bilateral exophthalmos	IV methylprednisolone followed by oral prednisolone	Resolution of orbital inflammation
“Checkpoint inhibitor-related myasthenia-myocarditis-myositis overlap syndrome in the orbit (2024)” (7)	Nivolumab	Ptosis and ophthalmoplegia	Oral prednisone 1 mg/kg daily	Resolution of ptosis and diplopia
	Ipilimumab and nivolumab	Ptosis, ophthalmoplegia, dyspnea, and generalized muscle weakness	A combination of IV immunoglobulin infusions, mycophenolate mofetil, pyridostigmine, and oral prednisone	Resolution of ptosis and diplopia
	Pembrolizumab	Rapid-onset bilateral ptosis and chemosis, ophthalmoplegia, and generalized weakness	IV methylprednisolone 1 g daily, IV immunoglobulin, oral pyridostigmine, and mycophenolate mofetil	Patient passed away before treatment effects could be assessed
“Ipilimumab-related orbitopathy: a case report (2022)” (10)	Ipilimumab	Right-sided proptosis with orbital inflammation, ophthalmoplegia, and esotropia	IV corticosteroid and immunoglobulin therapy	Resolution of inflammation and ophthalmoplegia
“Thyroid autoimmunity and ophthalmopathy related to melanoma biologic therapy” (2011) (13)	Ipilimumab	Eye pain, proptosis, and periorbital edema	IV and PO corticosteroid	Complete resolution of ocular symptoms
“Inflammatory Orbitopathy Associated With Ipilimumab” (2017) (11)	Ipilimumab	Bilateral proptosis, periorbital edema, upper eyelid retraction, and ophthalmoplegia	IV and PO corticosteroid	Complete resolution of ocular symptoms
“Rapid Development of Graves’ Ophthalmopathy After Treatment With Ipilimumab and Recurrence With Pembrolizumab in a Patient With Previously Treated Graves’ Disease” (2018) (8)	Ipilimumab and pembrolizumab	Bilateral proptosis, chemosis, diplopia, and ophthalmoplegia	IV and PO corticosteroid	Complete resolution of ocular symptoms
“Phase I/II trial of tremelimumab in patients with metastatic melanoma” (2009) (21)	Tremelimumab	Periorbital edema, conjunctival injection and chemosis, and proptosis	IV corticosteroids	Complete resolution of ocular symptoms
“Ipilimumab-induced Adenohypophysitis and Orbital Apex Syndrome: Importance of Early Diagnosis and Management” (2017) (14)	Ipilimumab	Vision loss, proptosis, and ophthalmoplegia	IV methylprednisolone	Resolution of vision loss, but persistent esotropia

Pembrolizumab is an additional immune checkpoint inhibitor potentially implicated in the activation of TED, and its mechanism of action entails blocking PD-1. One case of pembrolizumab use was associated with rapid development of Graves’ orbitopathy within 24 hours of its initiation, combined with elevated thyroid-stimulating hormone receptor antibodies, in a patient who had actually developed a similar presentation with ipilimumab use in the past (8).

The majority of reported cases of inflammatory orbitopathies in the setting of ICI use involved ipilimumab, a CTLA-4 inhibitor. One case involved complete ophthalmoplegia without other inflammatory signs, while thyroid function tests remained normal (9). In another patient, ipilimumab infusions triggered diffuse periorbital edema and ophthalmoplegia correlated with enlargement of extraocular muscles on imaging, with both lateral recti being most enlarged, distinct from the patterns of muscle enlargement seen most commonly in thyroid eye disease (10). Another patient who started ipilimumab as part of her cancer treatment developed proptosis, conjunctival chemosis and injection, and ophthalmoplegia along with extensive muscle and tendon enlargement on computed tomography imaging but was actually found to be hypothyroid (11). In many cases involving ipilimumab, imaging also revealed tendon-involving muscle enlargement, as opposed to the tendon-sparing form classically seen in TED. Case reports of inflammatory orbitopathies after ipilimumab use were associated with both TED activation and orbital syndromes that did not coincide with elevated thyroid function and antibody tests (12–14).

Finally, some reports of thyroid eye disease activation have been described in patients who underwent treatment with tremelimumab. One patient developed a hyperthyroid state, confirmed by serum testing, and orbital inflammation with a CAS of 6 (6). Another patient developed periorbital edema and proptosis associated with enlargement of extraocular muscles on imaging, correlating with elevated thyroid function tests consistent with hyperthyroidism (15).

## Discussion

This case report and literature review highlights the risk of developing orbital inflammation secondary to the use of immune checkpoint inhibitors. Previous studies have suggested that CTLA-4 is implicated in the pathogenesis of Graves’ disease and that PD-L1 expression is elevated in Hashimoto’s hypothyroidism (1, 16, 17). (18) Inhibiting PD-1 can also result in thyroid antibody production by B cells (2). Other studies have determined that there may be a direct connection between the increased T-cell activity caused by ICIs and the development of orbital myositis, while some papers theorize that there may be a process similar to that of IgG-4-related disease (14). The mechanism underlying thyroid dysfunction, thyroid eye disease, or any inflammatory orbitopathies after ICI use is not yet well understood. Newer research has demonstrated that patients with thyroiditis triggered by ICI use have significantly larger cell counts of T lymphocytes (19). Similar studies have shown that other cell lines tend to proliferate in the setting of ICI-associated thyroiditis as well, including monocytes and natural killer cells, although their role in the pathogenesis has not yet been specifically delineated (20). We suggest caution in using ICIs in patients with a history of thyroid eye disease as well as multi-disciplinary collaboration across oculofacial plastic surgery, endocrinology, and oncology to co-manage these patients. Further research should investigate the pathophysiology and mechanisms that underlie this phenomenon to better understand how to counsel patients who may be starting an ICI.
